# A Switchable High-Performance RF-MEMS Resonator with Flexible Frequency Generations

**DOI:** 10.1038/s41598-020-61744-2

**Published:** 2020-03-16

**Authors:** Zeji Chen, Xiao Kan, Quan Yuan, Tianyun Wang, Jinling Yang, Fuhua Yang

**Affiliations:** 1 Institute of Semiconductors, Chinese Academy of Sciences, Beijing, 100083 P. R. China; 2 Center of Materials Science and Optoelectronics Engineering, University of Chinese Academy of Sciences, Beijing, 100049 P. R. China; 3 State Key Laboratory of Transducer Technology, Shanghai, 200050 P. R. China

**Keywords:** Electrical and electronic engineering, Mechanical engineering

## Abstract

Resonators with multi-frequency generations at the device-level are highly desired in the future multi-band, reconfigurable, and compact wireless communications. In this work, a switchable radio frequency micro-electro-mechanical system (RF-MEMS) resonator with multiple electrodes is presented. The resonator is designed to operate at the whispering gallery modes (WGMs). Simultaneous excitations of the second to seventh modes with high *Q* values are implemented within a single device using a pair of electrodes for driving and sensing. For effective multi-frequency excitations, the electrode span angle is optimized. The frequencies of the 37 μm and 18 μm-radius resonators range from 53 MHz to 176 MHz and 112 MHz to 366 MHz, respectively. The *Q* values in each mode are over 10^4^. Moreover, with the multi-electrode configurations, the specific mode can be enhanced with other modes suppressed. A more than 6 dB improvement of the spectrum peak is realized and the high *Q* values are maintained. A comprehensive theory is built up to clarify the driving/sensing principles under different electrode configurations. Furthermore, the air damping is found to have a significant effect on *Q* values for resonator with high stiffness vibrating in all the WGMs. The *Q* values in vacuum have at least two times improvement. The high-performance switchable resonator could dramatically reduce the power consumption, simplify the processing circuits, and occupy less footprint, which has great potential applications in future advanced RF front end systems.

## Introduction

Future wireless communications, like 5^th^ generation (5 G)^1,2^, Internet of Thing (IOT)^3,4^, and tactile Internet^5^, urge for radio frequency (RF) front end transceivers with high operating frequencies, pronounced tunability and reconfigurability, reduced hardware redundancy and less power consumption^6^. Radio frequency micro-electro-mechanical system (RF-MEMS) technologies are emerging as an enabling solution to address the fast-growing demands of the advanced wireless communications^7–9^. MEMS resonators with remarkable characteristics of high *f* × *Q* products, high-level IC compatibility, small footprint, and low power consumption have great potential in communication applications, which are regarded as the key element to constitute the future RF front end transceivers^10–14^. Mechanically coupling high-*Q* MEMS resonators, MEMS filters with ultra-narrow passbands less than 0.1% and high stopband rejection over 50 dB have been demonstrated^15^, which are beneficial for the direct channel selection. Meanwhile, using MEMS resonators as frequency selection components, MEMS oscillators with low phase noise of −138 dBc/Hz @ 1 kHz^16^ and excellent long-term frequency stability of ±0.1 ppm^17^ have been implemented. The miniaturized MEMS oscillators are regarded as competitive alternatives of traditional off chip devices such as quartz crystals and surface acoustic wave (SAW) resonators^18^.

Multi-frequency devices are highly desired to meet the requirements of wider frequency coverage^19^. Although the state-of-art MEMS resonators have exhibited outstanding performance, the devices mainly provide a single frequency. Besides, the limited tuning range of MEMS resonators is not sufficient for multi-frequency outputs, for instance, the contour mode disk resonators have very limited frequency shifts of approximately 0.1% at VHF range^20^. For filters based on the single-frequency resonators, massive devices with individual RF switches have to be entailed to process numerous communication standards, which leads to larger space occupation and more power consumption^21^. In addition, oscillators based on the single-frequency resonators might need phase lock loop (PLL) circuits to meet the multi-frequency requirement, which results in complicated control circuits and enhanced phase noise^22^.

To solve this issue, significant work has been carried out and the multi-frequency RF modules have been realized at the chip-level. With resonators vibrating in the in-plane modes, such as contour or length extensional modes, a series of devices of different geometrical dimensions are fabricated in a single chip to form reconfigurable oscillators or multi-band filters^23–25^. Nevertheless, exploiting resonators with multi-frequency outputs within a single device would be preferable for a compact monolithic RF front end transceiver. Recently, a two-port Lithium Niobate MEMS resonator is developed, by which a self-coupled filter is proposed with two center frequencies of 500 MHz and 750 MHz. The corresponding fractional bandwidths are 2% and 3.9%, respectively^26^. However, the *Q* values of the resonator are not high enough, which restricts its applications as channel-selection RF transceivers.

The multiple-electrode structure was expected to be able to simultaneously excite different whispering gallery modes (WGMs) for multi-frequency outputs. However, the previous work mainly focused on the specific WGM excitation, or only few WGM excitations covering narrow frequency span with the multi-electrode arrangement^27,28^. For example, the multiple electrodes have been employed to drive the 2^nd^ and 3^rd^ WGMs of a single crystal silicon gyroscope, but the 3^rd^ mode with a reduced sensitivity is not desired^29^. In our reported work^30,31^, the simultaneous excitations of the 2^nd^ ~ 7^th^ WGMs for the disk resonators with a pair of electrodes were preliminarily demonstrated in experiment, but the principles of the multiple-mode excitations have not been clearly clarified and an extensive theoretical analysis for multiple-mode driving/sensing mechanisms was not yet given.

This work presents a high-performance switchable RF-MEMS disk resonator with multiple electrodes uniformly distributed along the circumference, experimentally and theoretically studied on the distinctive driving/sensing principle and energy dissipation mechanisms. The resonator vibrating in WGMs has outperforming *f* × *Q* products and multi-frequency outputs. A comprehensive theoretical model for the WGM resonator was built up, and the systematical measurements of the resonators with various designs were carried out to verify the theoretical analysis on the driving/sensing principle and energy dissipation mechanisms. Finally, a design guidance for the high-end resonator was provided. The proposed switchable resonator with flexible frequency generations can be adopted to different applications, such as multiple clock generations, multi-band channel-selection filters, and low insertion loss mixers. Employing a series of multi-frequency resonators of different dimensions, the wider operating frequencies can be covered and a compact communication system with dramatically reduced power consumption and much smaller footprint is expected.

## Resonator design and operation

### Mode shapes and resonance frequencies

**Figure 1:**
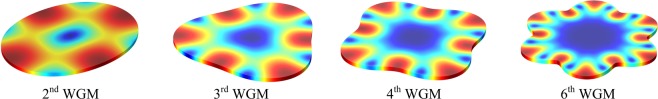
The simulated mode shapes of the 2^nd^, 3^rd^, 4^th^ and 6^th^ WGMs. The standing waves are formed at the edge of the disk, the vibration antinodes are uniformly distributed along the circumference with adjacent lobes moving out of phase.

The resonator is designed to operate in WGMs. The simulated mode shapes of WGMs are given in Fig. 1. As can be seen, the standing waves are formed at the edge of the disk. The vibration antinodes are uniformly distributed along the circumference with adjacent lobes moving out of phase. The characteristic of WGMs with multiple lobes is beneficial for the multi-frequency excitations at the device-level^32^. To have an optimized resonator design, the vibration displacement distribution should be quantified. The origin is set at the disk center and the coordinates *r* and *θ* are used. The fundamental equation for in-plane vibrations governed by the elastic theory can be written as the following format^33^:

1 $$\frac{E}{1-{\upsilon }^{2}}\cdot \nabla (\nabla \cdot \overrightarrow{u})-\frac{E}{2(1+\upsilon )}\cdot \nabla \times \nabla \times \overrightarrow{u}=\rho \frac{{\partial }^{2}\overrightarrow{u}}{\partial {t}^{2}},$$

where *E* is the Young’s modulus, *υ* is the Poisson’s ratio, and *ρ* is the density. The displacement vector $\overrightarrow{u}$ is associated with the scalar potential Φ and the vector potential $\overrightarrow{\Psi }$, which can be defined as^34^:

2 $$\overrightarrow{u}=\nabla \Phi +\nabla \times \overrightarrow{\Psi }.$$

Because of the in-plane vibration, $\overrightarrow{u}$ contains only *r-*coordinate and *θ-*coordinate components. To satisfy this condition, $\overrightarrow{\Psi }$ should only have a nonzero component *ψ* in z-coordinate^34^. Combining Eqs. (1) and (2), the potentials can be written as:

3 $$\begin{array}{c}\frac{{\partial }^{2}\Phi }{\partial {t}^{2}}=\frac{E}{\rho (1-{\upsilon }^{2})}\cdot {\nabla }^{2}\Phi ,\\ \frac{{\partial }^{2}\psi }{\partial {t}^{2}}=\frac{E}{2\rho (1+\upsilon )}\cdot {\nabla }^{2}\Psi .\end{array}$$

The solutions of Eq. (3) are:

4 $$\begin{array}{c}{\Phi }_{n}={M}_{n}{J}_{n}\left({k}_{n}\frac{r}{R}\right)\cdot \,\cos \,n\theta \cdot {e}^{j{\omega }_{n}t},\\ {\psi }_{n}={N}_{n}{J}_{n}\left({h}_{n}\frac{r}{R}\right)\cdot \,\sin \,n\theta \cdot {e}^{j{\omega }_{n}t},\end{array}$$

where *M*_*n*_ and *N*_*n*_ are constants, *J*_*n*_ is the Bessel function of the first kind, *R* is the radius of the disk resonator, *n* is the mode order, and *ω*_*n*_ refers to the angular resonance frequency of *n*^*th*^ mode, *k*_*n*_ and *h*_*n*_ are frequency-dependent parameters expressed as^34^:

5 $$\begin{array}{c}{k}_{n}={\omega }_{n}\frac{R}{\sqrt{\frac{E}{\rho (1-{\upsilon }^{2})}}},\\ {h}_{n}={\omega }_{n}\frac{R}{\sqrt{\frac{E}{2\rho (1+\upsilon )}}}.\end{array}$$

Substituting Eq. (4) into Eq. (2), the displacement components along the radial and tangential direction can be attained^34^:

6 $$\begin{array}{c}{X}_{n}=\left[{M}_{n}\frac{d}{dr}{J}_{n}\left({k}_{n}\frac{r}{R}\right)+\frac{n}{r}{N}_{n}{J}_{n}\left({h}_{n}\frac{r}{R}\right)\right]\cos \,n\theta ,\\ {Y}_{n}=\left[{M}_{n}\frac{n}{r}{J}_{n}\left({k}_{n}\frac{r}{R}\right)-{N}_{n}\frac{d}{dr}{J}_{n}\left({h}_{n}\frac{r}{R}\right)\right]\sin \,n\theta .\end{array}$$

As only the radial displacements lead to the variation of the capacitive gap, which generates the motional current, the tangential component is not discussed here. To have an intuitional description about the mode shape of WGMs, the radial displacement amplitude at an arbitrary point (*r*, *θ*) is normalized to attain the relative displacement distribution, thus, Eq. (6) can be rewritten as^35^:

7 $${X}_{{\rm{mode}}}=\left[\frac{d}{dr}{J}_{n}\left({k}_{n}\frac{r}{R}\right)+\frac{n}{r}\frac{{N}_{n}}{{M}_{n}}{J}_{n}\left({h}_{n}\frac{r}{R}\right)\right]\cos \,n\theta .$$

In essence, Eq. (7) describes the mode shape of WGMs. To depict the resonance frequency of the WGM, the boundary conditions should be considered. The normal stress as well as the shear stress at the circumference are zero, which are expressed as^36^:

8 $$\begin{array}{c}{{T}_{rr}|}_{r=R}=\frac{E}{(1-{\upsilon }^{2})}\frac{\partial {X}_{n}}{\partial r}+\frac{E\upsilon }{(1-{\upsilon }^{2})}\frac{{X}_{n}}{r}+\frac{E\upsilon }{(1-{\upsilon }^{2})}\frac{1}{r}\frac{\partial {Y}_{n}}{\partial \theta }=0,\\ {{T}_{r\theta }|}_{r=R}=\frac{E}{2(1+\upsilon )}\left[\frac{\partial {Y}_{n}}{\partial r}+\frac{1}{r}(\frac{\partial {X}_{n}}{\partial \theta }-{Y}_{n})\right]=0.\end{array}$$

Substituting Eq. (6) into Eq. (8), an equation only depending on *k*_*n*_ and *h*_*n*_ can be attained:

9 $$[\begin{array}{cc}{a}_{11} & {a}_{12}\\ {a}_{21} & {a}_{22}\end{array}][\begin{array}{c}{M}_{n}\\ {N}_{n}\end{array}]=0,$$

where

$$\begin{array}{c}{a}_{11}=\frac{E}{(1-{\upsilon }^{2})}\left\{{\frac{{d}^{2}}{d{r}^{2}}{J}_{n}\left({k}_{n}\frac{r}{R}\right)|}_{r=R}+\frac{\upsilon }{R}\frac{d}{dr}{{J}_{n}\left({k}_{n}\frac{r}{R}\right)|}_{r=R}+\upsilon \frac{{n}^{2}}{{R}^{2}}{J}_{n}({k}_{n})\right\}\cos \,n\theta ,\\ {a}_{12}=\frac{E}{(1-{\upsilon }^{2})}\left\{{n\frac{d}{dr}\left[\frac{1}{r}{J}_{n}\left({h}_{n}\frac{r}{R}\right)\right]|}_{r=R}+\upsilon \frac{n}{{R}^{2}}{J}_{n}({h}_{n})-\upsilon \frac{n}{R}\frac{d}{dr}{{J}_{n}\left({h}_{n}\frac{r}{R}\right)|}_{r=R}\right\}\cos \,n\theta ,\\ {a}_{21}=\frac{E}{2(1+\upsilon )}\left\{{n\frac{d}{dr}\left[\frac{1}{r}{J}_{n}\left({k}_{n}\frac{r}{R}\right)\right]|}_{r=R}-\frac{n}{R}\frac{d}{dr}{{J}_{n}\left({k}_{n}\frac{r}{R}\right)|}_{r=R}-\frac{n}{{R}^{2}}{J}_{n}({k}_{n})\right\}\sin \,n\theta ,\\ {a}_{22}=\frac{E}{2(1+\upsilon )}\left\{{\frac{{d}^{2}}{d{r}^{2}}{J}_{n}\left({h}_{n}\frac{r}{R}\right)|}_{r=R}-\frac{{n}^{2}}{{R}^{2}}{J}_{n}({h}_{n})+\frac{1}{R}\frac{d}{dr}{{J}_{n}\left({h}_{n}\frac{r}{R}\right)|}_{r=R}\right\}\sin \,n\theta .\end{array}$$

To have non-trivial solutions of *M*_*n*_ and *N*_*n*_, $[{a}_{ij}]$ should satisfy:

10 $$\det {[{a}_{ij}]}_{2\times 2}=0.$$

According to Eqs. (5) and (10), the values of *k*_*n*_ and *h*_*n*_ can be acquired, furthermore, the resonance frequencies of WGM can be determined as:

11 $${f}_{n}=\frac{{k}_{n}}{2\pi R}\sqrt{\frac{E}{\rho (1-{\upsilon }^{2})}}.$$

Using Eq. (11), the frequencies of each mode for the 37 μm radius resonators are summarized in Table 1.

**Table 1:** Resonance frequencies, calculated and measured RLC values of various modes for the resonators with 37 μm radius.

Mode (n)	Frequencies (MHz)	Resistance (kΩ)		Capacitance (F)		Inductor (H)	
		Calculated	Measured	Calculated	Measured	Calculated	Measured
2	53	765	835	4.30 × 10^−19^	3.94 × 10^−19^	20.50	22.35
3	83	754	915	2.38 × 10^−19^	2.15 × 10^−19^	15.73	17.43
4	107	1,286	1,345	10.7 × 10^−20^	8.97 × 10^−20^	20.61	24.56
5	130	1,755	1,704	6.39 × 10^−20^	6.58 × 10^−20^	23.39	22.71
6	154	2,150	2,310	4.18 × 10^−20^	3.89 × 10^−20^	25.30	27.21
7	176	3,008	3,050	3.15 × 10^−20^	3.11 × 10^−20^	25.87	26.23

### One-pair of electrode configuration

**Figure 2:**
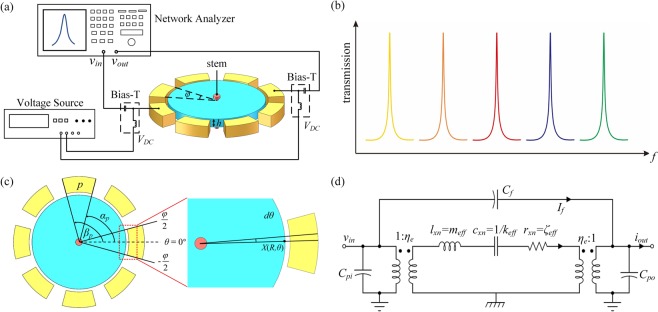
(**a**) The schematic of the one-pair of electrode configuration for the resonator with multiple electrodes. (**b**) With the one-pair of electrode configuration, simultaneous multi-frequency excitations within a single device can be achieved. (**c**) The maxima displacement is located at *θ* = 0° and the starting and ending angles of port *p* are *α*_*p*_ and *β*_*p*_, respectively. The displacement of an infinitesimal portion at the circumference in the electrode covered region is shown in the close-up schematic. (**d**) The equivalent circuit of the disk resonator.

This section details the derivation of the relationship between the motional current of different modes and the span angle. An explicit mathematical expression is given, by which the span angle is optimized for multi-frequency extractions. As shown in Fig. 2(a), the resonator is surrounded by eight uniformly distributed electrodes. An anchor stem is located at the center of the disk, which corresponds to the nodal region of the mode shape and achieves the minimum anchor loss. With a pair of electrodes used for driving and sensing, simultaneous excitations of multiple modes are realized within a single device, as shown in Fig. 2(b). In the one-pair of electrode configuration, the span angle of the electrode, *φ*, as shown in Fig. 2(a), is a critical parameter to determine the performance of multi-mode vibration. Utilizing the mode shape equation to analyze the motional currents of the resonator under external excitations is a practical method^37,38^.

When a DC bias voltage of *V*_*P*_ and an AC signal with the amplitude of *V*_*i*_ are applied to the resonator, an electrostatic force is generated, referred as^20^:

12 $$F={V}_{P}{V}_{i}\frac{\partial {C}_{i}}{\partial r}\cong {V}_{P}{V}_{i}\frac{{\varepsilon }_{0}Rh\varphi }{{d}_{0}^{2}},$$

where *ε*_0_ is the permittivity, *h* is the thickness of the resonator, *d*_0_ is the spacing gap, and $\frac{\partial {C}_{i}}{\partial r}$ is the resonator-electrode overlapped capacitance variation per unit displacement. As shown in Fig. 2(c), in the electrode overlapped region, the electrostatic force for an infinitesimal portion at the circumference can be expressed as:

13 $$dF={V}_{P}{V}_{i}\frac{{\varepsilon }_{0}Rh}{{d}_{0}^{2}}d\theta .$$

At resonance, the displacement induced by the external excitation is:

14 $$X={Q}_{n}\frac{F}{{k}_{re}},$$

where *X* denotes the induced displacement, *Q*_*n*_ is the quality factor of the *n*^*th*^ WGM, and *k*_*re*_ is the equivalent stiffness at the location where the electrostatic force is applied. Combing Eqs. (13) and (14), the displacement amplitude at the infinitesimal portion can be obtained^39^:

15 $$dX(R,\theta )=\frac{Q{V}_{P}{V}_{i}}{{k}_{eff}(R,\theta )}\frac{{\varepsilon }_{0}Rh}{{{d}_{0}}^{2}}d\theta ,$$

where *k*_*eff*_(*R*,*θ*) is the equivalent stiffness at the infinitesimal portion, which is associated with the equivalent mass at (*R*, *θ*):

16 $${k}_{eff}(R,\theta )={\omega }_{n}^{2}{m}_{eff}(R,\theta ).$$

The equivalent mass at any point can be calculated via dividing the total kinetic energy of the resonator by the half of the square of the velocity at the point as referred below:

17 $$\begin{array}{rcl}{m}_{eff}(r,\theta ) & = & \frac{K{E}_{total}}{\frac{1}{2}{[v(r,\theta )]}^{2}}\\  & = & \frac{\frac{1}{2}\int {[v(r{\prime} ,\theta {\prime} )]}^{2}dm}{\frac{1}{2}{[v(r,\theta )]}^{2}}\\  & = & \frac{\frac{1}{2}\int {[{\omega }_{n}X(r{\prime} ,\theta {\prime} )]}^{2}dm}{\frac{1}{2}{[{\omega }_{n}X(r,\theta )]}^{2}}\\  & = & \frac{\rho h{\int }_{0}^{2\pi }{\int }_{0}^{R}{[{X}_{\mathrm{mod}e}(r{\prime} ,\theta {\prime} )]}^{2}r{\prime} dr{\prime} d\theta {\prime} }{{[{X}_{\mathrm{mod}e}(r,\theta )]}^{2}}.\end{array}$$

It should be noted that as the elastic waves are transferring along the circumference, the contributions to the displacement at an infinitesimal portion contain not only the vibration induced by the electrostatic force at this location, but also the vibrations transferred from other points which behave as wave sources. Therefore, the total displacement of the portion should be the superposition of all the vibrations contributed by the entire region surrounded by the driving electrode. In addition, the displacement at (*R*,*θ*) contributed by electrostatic force at other location (*R*,*θ’*) can be written as^39^:

18 $$\frac{dX(R,\theta )}{dX(R,\theta {\prime} )}=\frac{{X}_{\mathrm{mod}{\rm{e}}}(R,\theta )}{{X}_{\mathrm{mod}{\rm{e}}}(R,\theta {\prime} )}.$$

Substituting Eqs. (16)–(18) into Eq. (15), and integrate all the vibrations from −*φ*/2 to *φ*/2, as shown in Fig. 2(c), the total displacement can be obtained:

19 $$X(R,\theta )=Q\frac{{V}_{P}{V}_{i}{\varepsilon }_{0}R}{\rho {{d}_{0}}^{2}{{\omega }_{n}}^{2}}\frac{{X}_{{\rm{m}}{\rm{o}}{\rm{d}}e}(R,\theta )}{{\int }_{0}^{2\pi }{\int }_{0}^{R}{[{X}_{{\rm{m}}{\rm{o}}{\rm{d}}e}(r{\rm{{\prime} }}{\rm{{\prime} }},\theta {\rm{{\prime} }}{\rm{{\prime} }})]}^{2}r{\rm{{\prime} }}{\rm{{\prime} }}dr{\rm{{\prime} }}{\rm{{\prime} }}d\theta {\rm{{\prime} }}{\rm{{\prime} }}}{\int }_{-\frac{\varphi }{2}}^{\frac{\varphi }{2}}{X}_{{\rm{m}}{\rm{o}}{\rm{d}}e}(R,\theta {\rm{{\prime} }})d\theta {\rm{{\prime} }}.$$

And the motional current at port *p* can be written as:

20 $${i}_{rp}={V}_{P}\frac{d{C}_{i}}{dt}={V}_{P}\frac{\partial {C}_{i}}{\partial r}\frac{\partial r}{\partial t}.$$

The maxima displacement is located at *θ*_0_ = 0, that is:

21 $${X}_{{\max }}=X(R,{\theta }_{0})=X(R,0).$$

According to Eq. (20), the motional current at the infinitesimal portion takes the form^39^:

22 $$d{i}_{rp}(R,\theta )=j{\omega }_{n}{V}_{P}\frac{{\varepsilon }_{0}Rh}{{{d}_{0}}^{2}}X(R,\theta )d\theta =j{\omega }_{n}{V}_{P}\frac{{\varepsilon }_{0}Rh}{{{d}_{0}}^{2}}\frac{{X}_{\mathrm{mod}e}(R,\theta )}{{X}_{\mathrm{mod}e}(R,{\theta }_{0})}X(R,{\theta }_{0})d\theta .$$

Substituting Eq. (19) into Eq. (22) and taking the integration from the starting angle *α*_*p*_ to the end angle *β*_*p*_ yields:

23 $${i}_{rp}=jQ\frac{{({\varepsilon }_{0}R)}^{2}h}{{\omega }_{n}{{d}_{0}}^{4}}\frac{{V}_{P}^{2}{V}_{i}}{\rho }\frac{{\int }_{-\frac{\varphi }{2}}^{\frac{\varphi }{2}}{X}_{{\rm{m}}{\rm{o}}{\rm{d}}e}(R,\theta )d\theta {\int }_{{\alpha }_{p}}^{{\beta }_{p}}{X}_{{\rm{m}}{\rm{o}}{\rm{d}}e}(R,\theta )d\theta }{{\int }_{0}^{2\pi }{\int }_{0}^{R}{[{X}_{{\rm{m}}{\rm{o}}{\rm{d}}e}(r{\rm{{\prime} }},\theta {\rm{{\prime} }})]}^{2}r{\rm{{\prime} }}dr{\rm{{\prime} }}d\theta {\rm{{\prime} }}}.$$

Substituting the mode shape Eq. (7) into Eq. (23), the relationship between *i*_*ri*_ and the span angle *φ* can be more explicit:

24 $${i}_{rp}={A}_{n}{\int }_{-\frac{\varphi }{2}}^{\frac{\varphi }{2}}\cos \,n\theta d\theta {\int }_{{\alpha }_{p}}^{{\beta }_{p}}\cos \,n\theta d\theta ,$$

where *A*_*n*_ is a constant which can be expressed as:

25 $${A}_{n}=jQ\frac{{({\varepsilon }_{0}R)}^{2}h}{\rho {{d}_{0}}^{4}{\omega }_{n}}{V}_{P}^{2}{V}_{i}\frac{{\left[\frac{d}{dr},{J}_{n},(,{k}_{n},),+,\frac{n}{R},\frac{{N}_{n}}{{M}_{n}},{J}_{n},(,{h}_{n},)\right]}^{2}}{{\int }_{0}^{2\pi }{\int }_{0}^{R}{[{U}_{{\rm{m}}{\rm{o}}{\rm{d}}e}(r{\rm{{\prime} }},\theta {\rm{{\prime} }})]}^{2}r{\rm{{\prime} }}dr{\rm{{\prime} }}d\theta {\rm{{\prime} }}}.$$

Moreover, for one-pair of electrode configuration, only one electrode is employed for sensing, thus, Eq. (24) can be written as:

26 $${i}_{rp}={A}_{n}{\left({\int }_{-\frac{\varphi }{2}}^{\frac{\varphi }{2}}\cos n\theta d\theta \right)}^{2}.$$

It can be indicated that in the *n*^*th*^ WGM, the motional current is only dependent on the span angle *φ* for a given excitation. Besides, when *φ* ranges from 0 to *π*/*n*, *i*_*ri*_ rises as *φ* increases until its maximum at *φ* = *π/n*; when *φ* ranges from *π*/*n* to 2*π*/*n*, *i*_*ri*_ drops with *φ* increasing and reaches zero at *φ* = 2*π/n*. The angles corresponding to the *i*_*rp, max*_ and *i*_*rp, min*_ of each mode are listed in Table 2.

**Table 2:** Span angles corresponding to the maximum and minimum motional currents in each WGM.

Mode (n)	Angles for *i*_*rp, max*_ (deg)	Angles for *i*_*rp, min*_ (deg)
2	90	180
3	60	120
4	45	90
5	36	72
6	30	60
7	25.7	51.4

**Figure 3:**
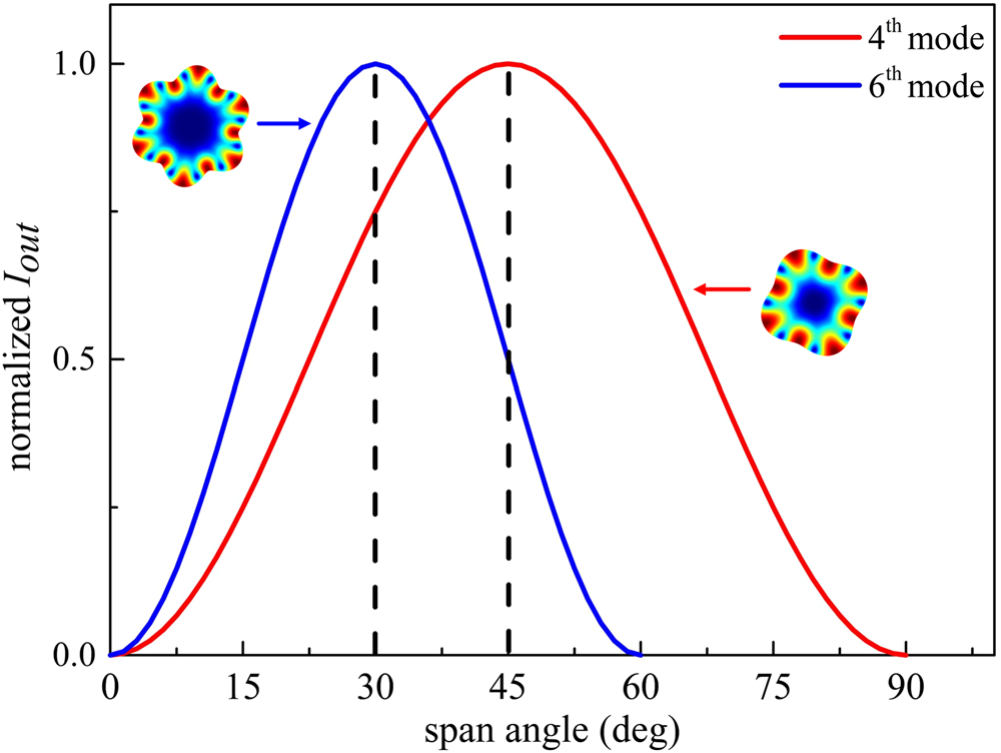
The normalized motional currents versus span angles for the 4^th^ and 6^th^ WGMs. When *φ* ranges from 0 to *π*/*n*, *i*_*ri*_ rises as *φ* increases until its maximum at *φ* = *π/n*; when *φ* ranges from *π*/*n* to 2*π*/*n*, *i*_*ri*_ drops with *φ* increasing and reaches zero at *φ* = 2*π/n*. The angles relating to the zero current tends to be smaller in the high-order modes, and a large span angle of the electrode leads to the suppression of high-order modes. On the contrary, a small span angle cannot provide sufficient electromechanical couplings for low-order modes. Therefore, a trade-off angle is required.

The normalized motional currents versus span angles for the 4^th^ and 6^th^ WGMs are plotted in Fig. 3.

As can be seen from Table 2, the angles relating to the zero current tends to be smaller as the mode order increases, a large span angle of the electrode leads to the suppression of high-order modes. Additionally, more electrodes are preferable for diverse electrode configurations, and the spacing between the electrodes should be optimized. However, a small span angle of the electrode cannot provide sufficient electromechanical couplings for low-order modes due to the limited transduction area. Therefore, to ensure the effective excitations of multiple modes in the single device, the span angle of the electrodes should be traded off. An angle between 30° and 40° is suitable. In this work, an angle *φ* = 34° is selected and the 2^nd^ to 7^th^ modes are simultaneously attained with high *Q* values in each mode.

With the optimized span angle of 34°, the equivalent motional resistance, inductor, and capacitance of the *n*^*th*^ mode of the resonator can be attained. The equivalent circuit is shown in Fig. 2(d), where *R*_*xn*_, *C*_*xn*_, and *L*_*xn*_ refer to the equivalent resistance, capacitance, and inductor of the resonator, *C*_*f*_ refers to the feedthrough capacitance, *C*_*pi*_ and *C*_*po*_ refer to the shunt static electrode-resonator overlap capacitances at the input and output ports, respectively. According to Eq. (20), the electromechanical coupling coefficient can be written as^39^:

27 $${\eta }_{e}=\frac{{i}_{rp}}{\dot{X}(R,{\theta }_{0})}={V}_{P}\frac{d{C}_{i}}{dt}={V}_{P}\frac{{\varepsilon }_{0}Rh}{{{d}_{0}}^{2}}{\int }_{-\frac{\varphi }{2}}^{\frac{\varphi }{2}}\frac{{X}_{\mathrm{mod}e}(R,\theta )}{{X}_{\mathrm{mod}e}(R,{\theta }_{0})}d\theta .$$

The equivalent damping *ζ*_*eff*_ can be expressed using the following formula:

28 $${\zeta }_{eff}(R,{\theta }_{0})=\frac{\sqrt{{k}_{eff}(R,{\theta }_{0}){m}_{eff}(R,{\theta }_{0})}}{{Q}_{n}}.$$

Thus, the equivalent motional resistance, inductor, and capacitance can be expressed as^20^:

29 $${R}_{xn}=\frac{{\zeta }_{eff}}{{{\eta }_{e}}^{2}},{L}_{xn}=\frac{{m}_{eff}}{{{\eta }_{e}}^{2}},{C}_{xn}=\frac{{{\eta }_{e}}^{2}}{{k}_{eff}}.$$

Using Eqs. (16), (17), (27)–(29), the equivalent RLC values of the 2^nd^–7^th^ modes can be obtained. For the 37 μm-radius disk resonator, the calculated equivalent RLC values are summarized in Table 1.

Given the mode shape of the resonator, the geometrical parameters, the electrode shape, and the excitation conditions, the output properties of the resonator can be attained.

### Multi-electrode configuration

**Figure 4:**
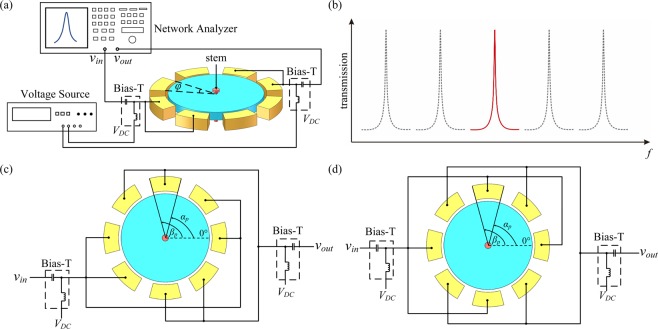
(**a**) The schematic of the multi-electrode configuration in the same device. (**b**) With the multi-electrode configuration, the specific mode can be excited with the other modes suppressed. (**c**) Multi-electrode configuration A of the WGM disk resonator: the two adjacent electrodes are both used as driving or sensing electrodes to excite the 2^nd^ WGM. (**d**) Configuration B: the adjacent electrodes are used as driving and sensing electrodes to excite the 4^th^ WGM.

Another advantage of the proposed resonator is the excitation of a specific mode with the other modes suppressed in the same device, as shown in Fig. 4(a). With multiple electrodes for driving and sensing, as shown in Fig. 4(b), the transduction area is substantially improved, which reduces the insertion loss of the desired mode. Meanwhile, some other modes can be suppressed as the electrodes located at different vibration regions extract anti-phase motional currents which can cancel out. The enhancement of the specific mode can widen the applications of the proposed resonator in advanced RF transceivers, which can alleviate the gaining requirement of the back-end amplifier circuits, consume less power as well as improve the system stability.

The aforementioned theory can be extended to analyze the motional currents of multi-electrode configuration. Given the configuration scheme as well as the starting and end angles of each electrode, the total currents can be calculated by summing up the output currents in each sensing electrode. Assuming that the number of sensing electrodes is *m*, the total current can be written as the following format:

30 $${i}_{total}={A}_{n}\mathop{\sum }\limits_{p=1}^{m}{\int }_{-\frac{\varphi }{2}}^{\frac{\varphi }{2}}\cos \,n\theta d\theta {\int }_{{\alpha }_{p}}^{{\beta }_{p}}\cos \,n\theta d\theta .$$

Equation (30) can be adopted for arbitrary electrode combinations. Two examples of the multi-electrode configurations are given to provide a comprehensive understanding of Eq. (30).

As shown in Fig. 4(c), in configuration A, the two adjacent electrodes are electrically connected for driving, while the next two electrodes are connected for sensing, and so on. The configuration is used to excite the 2^nd^ WGM. Pursuant to (30), the total motional current of each mode takes the form as:

31 $${i}_{n,total}={A}_{n}{\int }_{-\frac{\varphi }{2}}^{\frac{\varphi }{2}}\cos \,n\theta d\theta \cdot ({\int }_{-\frac{\varphi }{2}}^{\frac{\varphi }{2}}\cos \,n\theta d\theta +{\int }_{-\frac{\varphi }{2}+\frac{\pi }{4}}^{\frac{\varphi }{2}+\frac{\pi }{4}}\cos \,n\theta d\theta +{\int }_{-\frac{\varphi }{2}+\pi }^{\frac{\varphi }{2}+\pi }\cos \,n\theta d\theta +{\int }_{-\frac{\varphi }{2}+\frac{5\pi }{4}}^{\frac{\varphi }{2}+\frac{5\pi }{4}}\cos \,n\theta d\theta ),$$

which can be simplified as:

32 $${i}_{n,total}=\frac{4{A}_{n}}{{n}^{2}}{\sin }^{2}\frac{n\varphi }{2}\left([1+{(-1)}^{n}]+2\,\cos \,\frac{3}{4}n\pi \cdot \,\cos \,\frac{n\pi }{2}\right).$$

Equation (32) infers that all the odd modes are suppressed as the corresponding currents equal to zero. Besides, the currents of the 2^nd^, 4^th^, and 6^th^ WGMs are calculated to be $2{A}_{2}{\sin }^{2}\varphi$, 0, and $\frac{2{A}_{6}}{9}{\sin }^{2}3\varphi$, respectively. Therefore, for the 2^nd^ to 7^th^ WGMs, only the 2^nd^ and 6^th^ modes are excited. As there is a wide frequency interval between these two modes, the desired 2^nd^ mode is not distorted and the interference is confined. Moreover, compared with the one-pair of electrode configuration, the generated current in the 2^nd^ mode is significantly enhanced.

For configuration B as shown in Fig. 4(d), the adjacent electrodes are configured as the driving and sensing electrodes, respectively, which is used to excite the 4^th^ mode. Using Eq. (30), the total current can be determined as:

33 $${i}_{n,total}={A}_{n}\mathop{\sum }\limits_{i=1}^{4}{\int }_{-\frac{\varphi }{2}}^{\frac{\varphi }{2}}\cos \,n\theta d\theta {\int }_{-\frac{\varphi }{2}+\frac{\pi }{2}(i-1)}^{\frac{\varphi }{2}+\frac{\pi }{2}(i-1)}\cos \,n\theta d\theta ,$$

it can be simplified as:

34 $${i}_{n,total}=\frac{4{A}_{n}}{{n}^{2}}{\sin }^{2}\frac{n\varphi }{2}\cdot 2\,\cos \,\frac{n\pi }{2}\left(\cos \,\frac{n\pi }{2}+\,\cos \,n\pi \right).$$

It can be inferred from Eq. (34) that all the odd modes as well as the 2^nd^ and 6^th^ modes are suppressed as the corresponding currents are determined to be zero, while the current of the 4^th^ mode is ${A}_{4}{\sin }^{2}2\varphi$. Therefore, only the 4^th^ mode is excited and enhanced with the configuration B.

**Figure 5:**
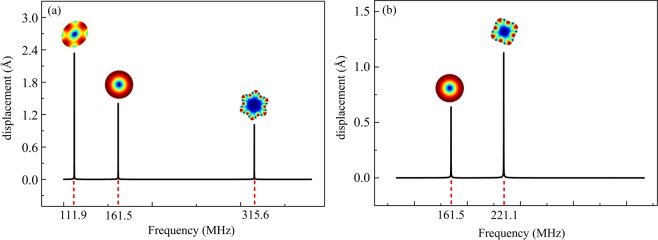
The simulated frequency response curves using FEA with the frequency sweeping over the 2^nd^ to 7^th^ WGMs. (**a**) the 2^nd^ and 6^th^ WGMs are excited with the configuration A and (**b**) the 4^th^ WGM is excited with configuration B. The contour mode is unavoidable due to its in-phase vibrations at the circumference.

To verify the above theoretical analysis, a finite element analysis (FEA) model is established. The external load is applied at the boundaries according to configuration A and B, and the frequency sweeping which covers the 2^nd^ to the 7^th^ mode is carried out to obtain the frequency responses. The simulated response curves for the configuration A and B are given in Fig. 5. As can be seen, the WGMs that can be excited under different configurations are consistent well with the theoretical deviations. It should be noted that the contour mode is unavoidable, since the vibrations at the circumference are all in-phase. Nevertheless, when the disk is scaled down, the frequency difference for the contour mode and the WGMs will be enlarged, which could greatly reduce the interference of the contour mode.

### Fabrication

The surface micromachining technology was used for the fabrication of the WGM disk resonators^40,41^. The 2.4 μm-thick LPCVD polysilicon with nearly zero-stress is deposited as a disk structural layer and a 70-nm capacitive gap is made by thermal oxidation. In addition, in terms of the nodal region distribution of the different WGMs, the anchor was set in the center of the disk. The resonator is released in 49% concentrated HF solution.

**Figure 6:**
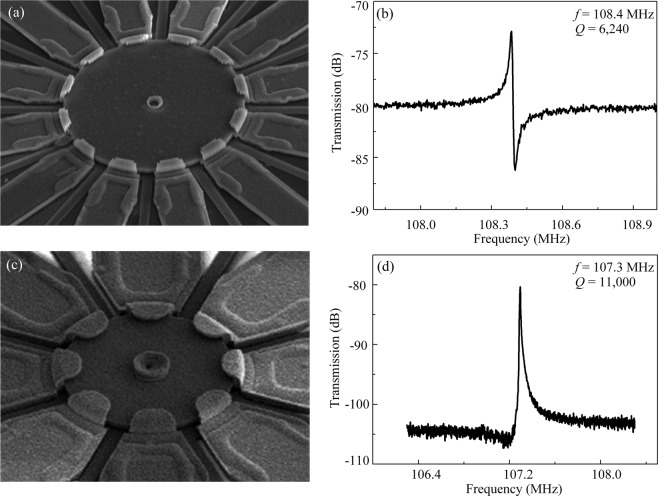
(**a**) The SEM photograph of disk with electrode angles of 20°. (**b**) The measured spectrum for 37 μm-radius disk resonator operating in the 4^th^ WGM with *θ* of 20°. The signal-to-background ratio (SBR) is 7 dB and the *Q* value is 6,240. (**c**) The SEM photograph of disk with electrode angles of 34°. (**d**) The measured spectrum for 37 μm-radius disk resonator operating in the 4^th^ WGM with *θ* of 34°, the SBR increases to 25 dB and the *Q* value reaches 11,000.

The 37 μm and 18 μm-radius disk resonators with multi-electrodes are produced on a (100)-oriented Si wafer. Figure 6(a,c) give the scanning electron microscope (SEM) photographs of the fabricated disk resonator with electrode span angles of 20° and 34°, respectively.

Agilent E5071C vector network analyzer was used for testing the *S* parameters and the Cascade probe station was employed for measurement at atmosphere. The disk is connected to the ground via the stem, a DC voltage and an AC signal were applied to the input electrode to excite the WGM modes, and another DC voltage was applied to the output electrode to generate the motional current. The bias voltage applied to the resonator is 10 V.

## Discussions

### Multiple-frequency output

In order to achieve multi-frequency outputs, the resonators are excited with the one-pair of electrode configuration, as shown in Fig. 2(a). Figure 6(b,d) give the frequency responses of 37 μm-radius disk with the different electrode span angles operating in the 4^th^ WGM. With the electrode angle of 20°, due to the large feedthrough, the signal-to-background ratio (SBR) is only 7 dB and the *Q* value is 6,240 (Fig. 6(b)). However, the SBR increases to 25 dB and the *Q* value reaches 11,000 for the 34° span angle (Fig. 6(d)). This result is consistent with the theoretical analysis, verifying that the span angle of the electrodes is critical for the multi-frequency generations in a single device and the optimized electrode span angle of 34° ensures excitation of the high-order WGMs with good SBR. The frequencies ranging from 53 MHz to 176 MHz with the *Q* values over 10^4^ in each mode are attained for the 37 μm disk resonator.

**Figure 7:**
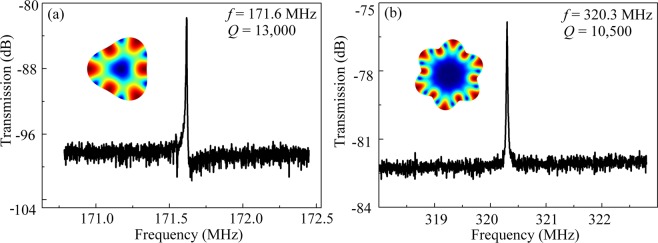
Measured spectra for the 18 μm-radius disk resonator with the span angle of 34°. In the 3^rd^ (**a**) and 6^th^ (**b**) mode, the resonance frequencies are 171.6 MHz and 320.3 MHz, with *Q* values of 13,000 and 10,500, respectively.

Furthermore, in order to achieve higher frequencies, an 18 μm-radius disk with the electrode span angles of 34° was fabricated. Figure 7 presents the frequency responses for an 18 μm-radius disk with the electrode span angles of 34° operating in the 3^rd^ WGM and 6^th^ WGM. The frequencies for the 3^rd^ WGM and the 6^th^ WGM are 171.6 MHz and 320.3 MHz, with the *Q* values 13,000 and 10,500, respectively.

The frequency and the *Q* values of the 2^nd^ to the 7^th^ modes of the 18 μm-radius disk resonators are summarized in Table 3. The maximum measured frequency is 366 MHz and the *Q* values for each mode are over 10^4^. The results indicate that when the resonator is scaled down, the higher frequencies can be obtained while the *Q* values are maintained high thus the *f* × *Q* products can be significantly improved. Therefore, the results manifest that the optimized span angle of the electrode can be adapted to the WGM-based disk resonators with different dimensions, which is in accordance with Eq. (26).

**Table 3:** Performance of the 18 μm disk resonators operated in WGMs.

Mode (n)	Frequencies (MHz)	*Q*
2	112	10,100
3	172	13,000
4	224	14,600
5	273	11,200
6	321	10,500
7	366	10,300

Additionally, utilizing the spectra peak values, the resonance frequencies, and the *Q* values, the measured motional resistance, inductor, and capacitance of each mode can be extracted via the following expression^42^:

35 $${R}_{m}=2{Z}_{0}({10}^{-\left(\frac{{G}_{prak}}{20}\right)}-1),\,{L}_{m}=\frac{{Q}_{n}{R}_{m}}{{\omega }_{n}},\,{C}_{m}=\frac{1}{{\omega }_{n}{Q}_{n}{R}_{m}}.$$

where *G*_*peak*_ is the transmission gain in decibels at the peak of the measured frequency characteristic; *Z*_0_ is the source or load resistance of the network analyzer (50 Ω). Utilizing Eq. (35), the measured RLC values of each mode for the 37 μm resonators are attained and compared with the calculated ones in Table 1. As can be seen, the measured and calculated values match well. The divergence could be attributed to the fabrication process tolerance and the parasitic effects, such as interconnect trace resistance, shunt static electrode-resonator overlap capacitances at the I/O ports, and so on. The high motional resistance of several hundred kΩ to several MΩ is ascribed to the limited electromechanical coupling coefficient of the capacitive transduction. There are several methods to decrease the motional resistance, such as decreasing the spacing gap^43^, filling the gap with high-*κ* solid dielectric materials^44,45^, and increasing the transduction area^46^.

To sum up, the multiple frequencies can be excited simultaneously by optimizing the electrodes span angles. Based on a series of resonators with different dimensions, a wide frequency range can be covered and a compact RF front end system can be expected.

### Multiple-electrode output

With the multiple electrodes configuration, as shown in Fig. 4(a), a special WGM can be excited and enhanced while the unwanted modes are suppressed. Two configurations are involved in the multiple-electrode scheme. For configuration A, as shown in Fig. 4(c), a pair of adjacent electrodes are connected for driving while the next pair of electrodes are connected for sensing, and so on. With this excitation scheme, the resonator vibrates in the 2^nd^ WGM. For configuration B, as shown in Fig. 4(d), two adjacent electrodes are used for driving and sensing, respectively, and the adjacent lobes are out of phase, thus, the resonator vibrates in the 4^th^ WGM.

**Figure 8:**
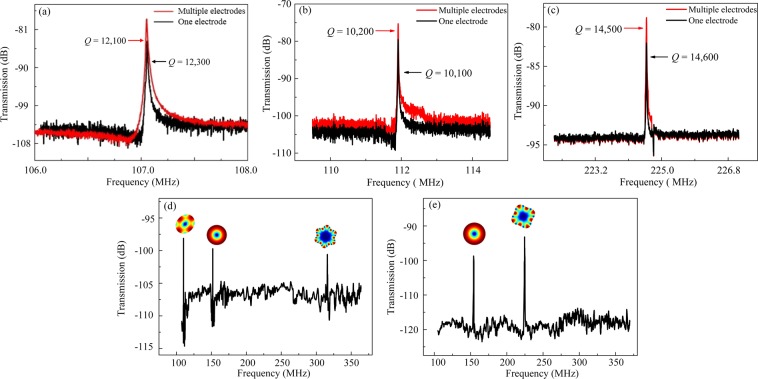
Comparison of frequency responses between the multi-electrode configurations and one-pair of electrode configurations for WGM disk resonators with different dimensions: the 4^th^ WGM of the 37 μm-radius disk (**a**), the 2^nd^ WGM of the 18 μm-radius disk (**b**), and the 4^th^ WGM of the 18 μm-radius disk (**c**). With the multi-electrode configurations, the *Q* values remain high and the spectra peaks are improved. Besides, the motional resistance can be effectively reduced. The measured wide frequency span (from 107 to 370 MHz) transmission spectra of multi-electrode configuration A (**d**) and configuration B (**e**) for the 18 μm-radius device are in accordance with the simulated results in Fig. 5.

The spectra of the 4^th^ WGM driven by the multiple-electrode configuration and the one-pair of electrode configuration for a 37 μm-radius disk are shown in Fig. 8(a). When the resonator was driven with the multiple-electrode configuration, the peak value of the multi-electrode configuration is more than 6 dB higher than that with the one-pair of electrode configuration. Moreover, there is no significant attenuation for the *Q* values. Besides, when the one-pair of electrode configuration is switched to the multi-electrode configuration, a larger transduction area is obtained. According to Eq. (35), the motional resistances decreases from 1.13 MΩ to 560 kΩ.

Similarly, the spectra for an 18 μm-radius disk resonator with the multi-electrode connections were shown in Fig. 8(b,c). With multiple-electrode configuration A, the resonator vibrates in the 2^nd^ WGM at a frequency of 111.9 MHz (Fig. 8(b)). For multiple-electrode configuration B, a resonance frequency of 224.6 MHz is obtained for the 4^th^ WGM (Fig. 8(c)). When the one-pair of electrode configuration is switched to the multi-electrode configuration, the *Q* values in both spectra remain high, besides, the motional resistances of the 2^nd^ and 4^th^ WGM reduce from 1.44 MΩ to 770 kΩ, and from 944 kΩ to 537 kΩ, respectively.

The measured wide frequency span (from 107 to 370 MHz) transmission spectra of configuration A and configuration B for the 18 μm-radius disk resonator are given in Fig. 8(d,e), respectively. As can be seen, the experimental results are consistent with the simulations shown in Fig. 5. The multi-electrode configurations can enhance the desired mode, suppress the unwanted modes over a wide frequency span, and substantially improve the flexibility of the frequency generations for the proposed resonator.

Above all, a special WGM of the multi-electrode resonator can be exclusively stimulated with a reduced insertion loss and high *Q* values. More multi-electrode configurations can be adopted for excitations of the other WGMs. In essence, with flexible electrode connection configurations, simultaneous excitations of multiple modes and special frequency generations can all be implemented in one device, which is beneficial for simplifying the wireless communications.

### Loss mechanisms

The experimental results have demonstrated that the high *Q* values could be maintained when the resonators are switched from the low-order modes to the high-order modes. This part attempts to provide a deep understanding about the loss mechanisms of the WGM based resonators.

The *Q* of the resonator is greatly affected by the different loss mechanisms, which can be categorized into air damping^47^, anchor losses^48^, surface losses^49^, thermoelastic damping (TED)^50^, phonon-phonon interactions^51^, and phonon-electron interactions^51^. The *Q*_*l*_ determined by each energy loss mechanisms contributes to the total *Q* of a micromechanical resonator according to the expression:

36 $$\frac{1}{Q}=\sum _{l}\frac{1}{{Q}_{l}},$$

where *l* refers to any one loss mechanism.

**Figure 9:**
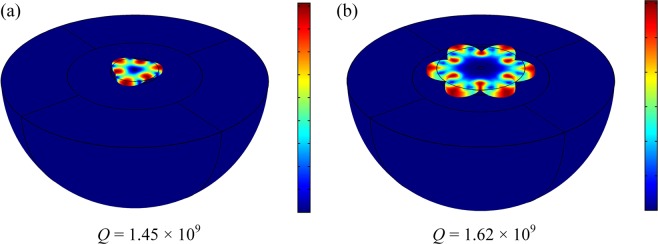
The simulated *Q* values of the 3^rd^ (**a**) and 6^th^ (**b**) WGMs for the 18 μm-radius resonator. The extended nodal region in high-order modes is beneficial for a higher *f* × *Q* product.

For the WGM disk resonator, as indicated by Fig. 1, the WGMs have much smaller motion at the center of the disk resonator compared with the contour mode. Besides, the nodal regions of the high-order WGMs extend towards the circumference of the disk resonator, which further reduces the anchoring loss and results in high *Q* values in the high-order WGMs. To verify this assumption, as shown in Fig. 9(a,b), the anchor loss of the disk operating in different WGMs is simulated with the FEA model. The perfect match layers (PMLs) are used to absorb the dissipated elastic waves from the anchor to the substrate^52^. The simulated *Q* values of the 3^rd^ and 6^th^ WGMs are 1.45 × 10^9^ and 1.62 × 10^9^, respectively. The extremely high *Q* values are attributed to the assumption that the anchor loss is the exclusive energy dissipation in the FEA model. The simulated results testify that the extended nodal region contributes to the high *Q* values at the high-order WGMs. Therefore, higher *f* × *Q* products can be achieved in the high-order modes. On the contrary, for the contour mode disk resonators, the *Q* values of the high order modes dramatically degrade due to severe anchor dissipation^20^.

**Figure 10:**
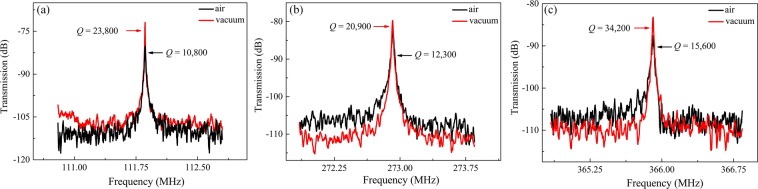
The frequency responses of the 2^nd^ (**a**), 5^th^ (**b**), and 7^th^ (**c**) modes for the 18 μm-radius disk resonator in vacuum and atmosphere. The effect of air damping on *Q* values is significant even for high-order WGMs.

To clarify the mechanical energy loss mechanism of the WGMs, the air damping effect of the resonator has been studied. With the operating pressure down to 9 × 10^−4^ Torr, the *Q* values achieve nearly 100% enhancement in each mode. As depicted in Fig. 10(a), for an 18 μm-radius resonator operating in the 2^nd^ mode, the *Q* factors increase from 10,800 at atmosphere to 23,800 in vacuum and the frequency spectrum peak value is improved. As shown in Fig. 10(b,c), the clear enhancements of *Q* values from 12,300 to 20,900 and from 15,600 to 34,200 are obtained for the 5^th^ and 7^th^ WGMs, which indicates that the air damping effect is significant even for the high-order WGMs with high stiffness, in contrary to the conventional tendency that the high-order bulk acoustic modes are insensitive to the air damping^20,53^. Therefore, although the WGM-based resonators have exhibited high *Q* values in atmosphere, the air damping cannot be neglected. It is necessary to package the device in vacuum for better performance.

## Conclusion

In this work, a switchable high-performance disk resonator operating in the WGMs is demonstrated. Multiple electrodes are distributed uniformly along the circumference, which allows for flexible electrode configurations in one device.

The mechanism of the multi-mode excitations in a single device is systematically studied. With the one-pair of electrode configuration as well as the optimized electrode span angle, the 2^nd^ to 7^th^ WGMs are simultaneously excited with the frequencies range from 53 MHz to 366 MHz and the *Q* values over 10^4^ in each mode. It would be a substantial step for multi-frequency generations.

A comprehensive theory was established for different electrode configurations to explore different applications. Multi-electrode configurations drive the specific WGMs with the improved spectra peaks and the reduced motional resistances, and suppress the other WGMs. The multi-electrode configurations widen the applications of the resonator.

The air damping effect is critical even for high-order WGMs with high stiffness. The *Q* values in vacuum for all the WGMs of the disk resonator have at least two times improvement, therefore, vacuum package is required for better performance.

Above all, the proposed resonator with high *f* × *Q* products and flexible driving/sensing schemes paves the way to the advanced multi-band and reconfigurable wireless communication systems with compact architectures, effectively reduced power consumption and smaller footprint.
